# Post-tonsillectomy pain after using bipolar diathermy scissors or the harmonic scalpel: a randomised blinded study

**DOI:** 10.1007/s00405-017-4451-9

**Published:** 2017-02-17

**Authors:** Linn Arbin, Mats Enlund, Johan Knutsson

**Affiliations:** 1 Dept of Otorhinolaryngology, Västerås Central Hospital, 721 89 Västerås, Sweden; 2 Dept of Anaesthesiology, Västerås Central Hospital and Centre for Clinical Research, Uppsala University, County hospital, 721 89 Västerås, Sweden; 3 Dept of Otorhinolaryngology, Västerås Central Hospital and Centre for Clinical Research, Uppsala University, County hospital, 721 89 Västerås, Sweden

**Keywords:** Tonsillectomy, Bipolar diathermy, Harmonic scalpel, Postoperative pain

## Abstract

**Objective:**

To compare the postoperative pain following bipolar diathermy scissors tonsillectomy (higher temperature dissection) with harmonic scalpel tonsillectomy (lower temperature dissection).

**Methods:**

Sixty patients aged 7–40 years planned for tonsillectomy with no other concurrent surgery were randomised to either bipolar diathermy scissors or harmonic scalpel as surgical technique. Blinded to the surgical technique, the patients recorded their pain scores (VAS, 0–10) at awakening and the worst pain level of the day in the postoperative period. All intake of pain medication was also recorded.

**Results:**

No statistically significant differences were found between the two groups regarding postoperative pain levels or consumption of pain medication.

**Conclusion:**

Usage of the harmonic scalpel does not render less postoperative pain following tonsillectomy when compared with usage of the bipolar diathermy scissors.

## Introduction

Many children and adults are affected by recurrent tonsillitis or tonsillar hypertrophy with upper airway obstruction. In these cases, tonsillectomy is often performed. The surgery causes considerable postoperative pain, often lasting more than a week after the procedure [1]. Tonsillectomy is often performed as day-case surgery, which increases the demands of a satisfactory postoperative pain control and a low risk of early postoperative bleeding.

The use of hot dissection techniques is associated with low rates of early postoperative bleeding and are therefore useful in day-case surgery. There are different hot dissection techniques including bipolar diathermy, harmonic scalpel, coblation and monopolar cautery [2].

The bipolar diathermy scissors coagulate at the same time as cutting, by applying an electrical current between the legs of the scissor. When using the bipolar diathermy scissors, the temperature in the tissue reaches 150–600 °C [3].

The effect that the harmonic scalpel exerts comes from a hook that vibrates at ultrasonic frequency (55.5 kHz) at a distance of 50–100 μm. The rapid vibrations build up a pressure in the tissue, causing the tissue to divide. The energy that is transmitted to the tissue denatures proteins and coagulates blood vessels, causing minimal tissue damage [4, 5]. In comparison with the bipolar diathermy scissors, the harmonic scalpel results in considerably lower tissue temperatures of 60–100 °C [5].

In theory, the harmonic scalpel causes less tissue damage than the bipolar diathermy scissors, by transmitting less energy to the surrounding tissues and thereby causing less pain after the operation, but previous studies are contradictory [6–10].

The aim of the study was to determine whether the choice between the two surgical instruments, which work at different temperatures, affects the patients’ postoperative pain. The hypothesis was that the harmonic scalpel renders less postoperative pain than the bipolar diathermy scissors.

## Materials and methods

The power analysis was based on the data distribution from an initial pilot study and a mean difference between the groups of 2.0 on the visual analogue scale (VAS), which was the smallest difference that was considered clinically relevant. The power analysis was performed with *α* = 0.05 and a power of 80%. Taking probable dropouts in mind, a total of 60 patients (30 in each group) were to be included in the study.

The Regional Ethical Review Board in Uppsala approved the study (Ref. Dnr 2011/303).

The patients were recruited from the department’s day-care unit and were given verbal and written information about the study during a clinic visit or by the surgeon on the morning of the day of surgery. Patients who fulfilled the inclusion criterion were eligible to participate in the study.

The inclusion criterion was:

- Patients between 7 and 40 years of age, scheduled for tonsillectomy for recurrent tonsillitis or upper airway obstruction due to tonsillar hypertrophy.

Exclusion criteria:

- Any concurrent surgical procedure, including adenoidectomy or insertion of transmyringeal ventilation tubes.
- Acute tonsillitis.
- Previous peritonsillar abscess.
- Inability to understand instructions (due to language barriers or impaired mental function).
- Bleeding disorder.
- Hypersensitivity/allergy to paracetamol or ibuprofen.

If the patient or caregiver accepted inclusion, the surgeon as well as the patient or caregiver signed the informed consent form. In total, 60 patients were included.

The patients were given oral oxycodone 0.20–0.30 mg/kg (<30 kg 5 mg, 30–60 kg 10 mg, >60 kg 20 mg), 30 min before the operation. Children who could not swallow tablets were given liquid oxycodone 1 mg/ml, 0.1 mg/kg = 0.1 ml/kg.

Children >30 kg and adults were anaesthetised with target-controlled infusions (TCI) of propofol and remifentanil. The initial targets were 6–7 µg/ml for propofol and 6–7 ng/ml for remifentanil, and the maintenance targets were 2–3 µg/ml and 2–3 ng/ml, respectively.

Children ≤30 kg were also anaesthetised with propofol and remifentanil with dosages corresponding to those above, but without the TCI function.

Anaesthesia induction could also be performed with sevoflurane inhalation via mask where the child refused an intravenous line. If so, anaesthesia was continued with propofol and remifentanil as soon as an intravenous line was introduced. All patients were intubated.

The theatre nurse performed the randomisation by opening the non-transparent envelope with the lowest remaining randomisation number. The content of the letter stated which of the two surgical techniques was to be used. The surgeons had training in the use of both surgical techniques.

The harmonic scalpel was set on a frequency of 55.5 kHz. The effect of the generator was set on power setting 3 (on the instrument’s scale of 1–5, where five cuts with the most power and thereby gives least coagulation) as previously reported [8]. When operating with the bipolar diathermy scissors, the energy was set at 16 W. After removal of the first tonsil, a cotton swab was placed in the tonsillar fossa, while proceeding with the contralateral tonsil. The cotton swab was then removed and if further haemostasis was needed on either side, a cotton swab was used for compression for 5 min in the tonsillar fossa. If the patient was in need of further haemostasis, the surgeon used bipolar diathermy. This was then noted in the surgical form including the total number of seconds the bipolar diathermy had been used for haemostasis.

Perioperatively, all patients were given glycopyrron (ca 5 µg/kg), fentanyl (2–3 µg/kg), betamethasone (4 mg/ml 1 ml <30 kg, 1.5 ml 30–50 kg and 2 ml >50 kg), intravenous paracetamol (10 mg/ml 1–1.5 ml/kg) and ondansetron 2 mg/ml 1 ml <30 kg, 1.5 ml 30–50 kg and 2 ml >50 kg. Furthermore, the surgeon, after removal of the tonsils, injected 5–10 ml of bupivacaine (5 mg/ml) in the anterior and posterior palatal arch.

After the operation, the surgeon filled in the surgical form specifying the surgical method and method of haemostasis. The surgical form was then put in a binder in the surgical unit for safe storage. The surgical method that had been used was kept blinded to the patient (and caregiver in case of children) as well as for the staff of the postoperative unit to keep the study double-blinded.

The patients stayed at least 3 h at the postoperative observation unit. Paracetamol 10 mg/kg and ibuprofen 5 mg/kg were given 4–6 h after the initial intravenous dose of paracetamol during surgery. Morphine was given intravenously if necessary and was noted on the reply form. Furthermore, patients received tablets of oxycodone in the same dosage as preoperatively to take at home in the evening and an additional prescription of oxycodone to pick up if necessary. Adults were prescribed oxycodone 5 mg one to four tablets twice a day and children were prescribed according to their weight: <30 kg, one tablet twice a day; 30–60 kg, one to two tablets twice a day; >60 kg, the same dose as adults.

Postoperatively, the patients were asked to do a VAS scoring 30 min and 3 h after the operation. Instructions were given to the patient or caregiver before discharge on how to use the provided VAS-ruler and how to fill out the reply form. An ENT nurse called the patient or caregiver the day after surgery to make sure that the reply form was being filled out. The patient then filled out the reply form daily for 10 days, including the amount and sort of pain medication used, the degree of postoperative throat pain (by using the VAS) at awakening (before intake of pain medication) and the worst level of pain during that day. The patients also noted any additional contacts with health care for any reason and sent in the form. The ENT nurse called the patient approximately 20 days after surgery to remind the patient to send in the reply form, if it had not already been received.

The results were analysed using SPSS 23.0. Continuous variables from the VAS scale were analysed using the Mann–Whitney *U* test and dichotomous variables were analysed using Fisher’s exact test.

## Results

Forty-one patients returned the reply form. One patient in the harmonic scalpel group had been included in the study by mistake (higher age than the inclusion criterion stated) and was thus withdrawn from the analysis. In total, the results from 40 patients were analysed.

In the harmonic scalpel group [*n* = 22 (8 males and 14 females)], the mean age was 20.27 years (SD 6.96; range 9–36). In the bipolar diathermy scissors group [*n* = 18 (4 males and 14 females)], the mean age was 21.83 years (SD 5.77; range 14–37). There was no statistical difference between the groups for age at surgery (*p* = 0.199).

In the harmonic scalpel group, 8 patients were operated due to chronic tonsillitis, 11 due to recurrent tonsillitis and 3 due to upper airway obstruction. In the bipolar diathermy scissors group, six patients were operated due to chronic tonsillitis, nine due to recurrent tonsillitis and three due to upper airway obstruction.

No statistical significance was found (*p* = 0.95) regarding the number of seconds bipolar diathermy was used for haemostasis after the tonsillectomy—21.2 s (SD 21.9) in the bipolar diathermy scissors group and 17.1 s (SD 12.8) in the harmonic scalpel group. Also, there was no difference between the groups when analysing only the patients for whom bipolar diathermy had been used for haemostasis, to exclude its possible interference with the results.

In both groups, the patients scored the highest pain levels on the VAS on the fourth postoperative day. The mean VAS scores for pain at awakening and worst level of pain during the day for postoperative days 1, 4 and 7 are presented in Table 1 and 2.

**Table 1:** Pain at awakening in the morning (mean VAS-score)

	Postop day 1	Postop day 4	Postop day 7
Harmonic scalpel	5.17 (CI 4.36–5.98)	6,47 (CI 5.32–7.62)	5,62 (CI 4.46–6.77)
Bipolar diathermy scissors	5.69 (CI 4.42–6.70)	7.27 (CI 6.59–7.94)	6.70 (CI 5.61–7.79)
*p* value	*p* = 0.33	*p* = 0.52	*p* = 0.36

**Table 2:** Worst level of pain during the day (mean VAS score)

	Postop day 1	Postop day 4	Postop day 7
Harmonic scalpel	5.65 (CI 4.87–6.43)	6.75 (CI 5.65–7.84)	6.08 (CI 4.94–7.21)
Bipolar diathermy scissors	6.47 (CI 5.47–7.47)	7.50 (CI 6.85–8.15)	7.03 (CI 5.98–8.08)
*p* value	*p* = 0.32	*p* = 0.53	*p* = 0.65

There was no significant difference in the postoperative pain between the two groups at any time of the follow-up. The mean difference of the VAS scores between the two groups was in each comparison less than 1.0 units.

Also, there was no significant difference between the groups regarding the reported use of analgesics on postoperative days 1, 4 and 7 (*p* values ranging from 0.41 to 0.93).

The reported number of health-care contacts, for any reason, did not differ between the groups. Two patients in each group contacted health-care providers due to signs of postoperative bleeding, none of which needing surgical intervention.

## Discussion

Post-tonsillectomy pain is a considerable problem leading to suffering and unplanned health-care contact [1]. The use of the harmonic scalpel could be advantageous from a theoretical point of view regarding postoperative pain, since it renders less energy to the surrounding tissues than other hot dissection techniques. Postoperative pain following other kinds of surgery have been reported to be lowered by the use of the harmonic scalpel [11–13]. Previous reports on the possible pain-reducing effect by the use of the harmonic scalpel for tonsillectomy show contradictory results [9, 14, 15].

For the pain assessment in the present study, the visual analogue scale (VAS) was used. In addition, the amount of analgesia used was recorded as a surrogate measure of pain. Psychometric studies of the VAS have shown that it has good validity and reliability (test–retest *r* = 0.95) [16]. The VAS has been used previously for the assessment of postoperative pain in adults and children following tonsillectomy [1].

The present study found mean VAS scores for the worst level of pain between 5.65 and 7.50 which is equivalent with the VAS scores found in a previous study of the harmonic scalpel and electrocautery for tonsillectomy on patients aged 6–15 years [10]. That study reported worst pain mean VAS scores between 6.2 and 6.9 for the first 6 days after tonsillectomy, with no major pain differences in the first six postoperative days. In the present study, the patients estimated the highest VAS scores on the fourth postoperative day regardless of which technique had been used.

No differences were found between the two groups regarding postoperative pain at any time. The use of potent analgesics postoperatively could in theory have suppressed the pain levels in both groups resulting in reduced pain score differences between the two groups, masking possible differences in the pain that the two surgical techniques render. We consider the risk of such an effect as low, since both groups scored relatively high VAS values and at a level previously reported after controlled studies of tonsillectomy [6, 10].

The possibility of having made a type II error, i.e. the risk of not detecting a clinically relevant difference between the groups due too small sample size, has been considered. Since the *p* values were relatively high, we consider this risk to be low. Other studies with smaller patient samples have found significant pain differences when testing the harmonic scalpel versus cold dissection techniques. In a study of 28 patients, Collison et al. found less early postoperative pain after the use of the harmonic scalpel [17]. Akural et al. found similar results in a study of 32 patients, but an increased pharyngeal pain in the later postoperative phase compared to cold dissection technique [18].

The harmonic scalpel versus electrocautery has previously been studied in a few clinical trials regarding tonsillectomy. One non-randomised study comparing postoperative pain after the use of a harmonic scalpel versus electrocautery found no difference between the groups, except when rescue usage of electrocautery was needed to stop bleeding in the harmonic scalpel group, where more pain was noted [19]. Whether monopolar or bipolar electrocautery was used was not reported making comparison with the present study difficult. The harmonic scalpel has previously been studied versus monopolar cautery specifically. No significant difference between the two groups was found regarding postoperative pain [5].

Previous studies that most resemble the present study compare harmonic scalpel tonsillectomy with bipolar diathermy tonsillectomy in the paediatric age group. Kemal et al. found lower pain levels in the harmonic scalpel group [9]. As in the present study, Leaper et al. on the other hand found no advantage of using harmonic scalpel [10]. On the contrary, the patients operated with the harmonic scalpel had increased pain compared with the bipolar diathermy technique. The main difference was seen when analysing the worst pain of the day. Patients undergoing concurrent adenoidectomy were not excluded from those two studies. In the present study, we chose not to include patients undergoing other concurrent surgical interventions, since it might be difficult for a patient to denominate the origin of the pain after concurrent surgeries. The same difficulties may have affected a study including 21 adults undergoing tonsillectomy, using harmonic scalpel on one side and bipolar diathermy on the other. The study did not find any differences regarding postoperative pain between the sides at any time point [20].

The clinical application of the present results is that none of the two surgical instruments is superior to the other in terms of postoperative pain. Regarding other differences between the surgical instruments, we did not find any reported health economy analyses on the use of different kinds of surgical instruments for tonsillectomy. Considering the result of the present randomised study and the lack of other studies showing an advantage of the harmonic scalpel for tonsillectomy, the use of the harmonic scalpel can be questioned from an economical point of view. The single-use disposable unit, used in this study, costs approximately 90 Euro per patient, compared with the manyfold lower cost of using multiple-use instruments.

## Conclusion

Tonsillectomy using the harmonic scalpel did not result in reduced postoperative pain compared with bipolar diathermy. This double-blinded randomised study found no differences in VAS scores between the groups; neither was there any difference between the groups regarding the amount of analgesics used postoperatively, which reinforces the results.
